# Obesity After Kidney Transplantation—Results of a KTx360°Substudy

**DOI:** 10.3389/fpsyt.2020.00399

**Published:** 2020-05-08

**Authors:** Mariel Nöhre, Elisabeth Schieffer, Alexander Hanke, Lars Pape, Lena Schiffer, Mario Schiffer, Martina de Zwaan

**Affiliations:** ^1^Department of Psychosomatic Medicine and Psychotherapy, Hannover Medical School, Hannover, Germany; ^2^Project Kidney Transplantation 360° (NTX360°), Hannover Medical School, Hannover, Germany; ^3^Department of Sports Medicine, Hannover Medical School, Hannover, Germany; ^4^Department of Pediatric Kidney, Liver and Metabolic Diseases, Hannover Medical School, Hannover, Germany; ^5^Department of Nephrology and Hypertension, Hannover Medical School, Hannover, Germany; ^6^Department of Nephrology and Hypertension, University Hospital Erlangen, Erlangen, Germany

**Keywords:** obesity, overweight, kidney transplantation, renal transplantation, kidney functioning

## Abstract

**Objective:**

There is solid evidence that kidney transplant (KTx) patients are susceptible to weight gain after transplantation. Post-transplantation obesity [body mass index (BMI) ≥ 30 kg/m^2^] seems to be associated with higher risks of hypertension, dyslipidemia, diabetes mellitus, and cardiovascular events, while there are contradicting findings regarding the association between obesity and mortality, graft failure after transplantation as well as other variables. We aimed to evaluate the course of weight after KTx and to assess the prevalence of post-transplant obesity in a large sample of German KTx patients. Further, we focused on potential associations between weight gain, obesity, and BMI after transplantation with sociodemographic, medical, psychological [levels of anxiety and depression measured with the Hospital Anxiety and Depression Scale (HADS)], and donation-specific variables.

**Methods:**

In a structured post-transplant care program 433 KTx patients were evaluated at Hannover Medical School. Information on the pre-transplant body weight/dry weight of dialysis patients was taken from the electronic patient charts. At post-transplant assessment body weight was measured in the transplant center. For statistical analyses, descriptive statistics, analyses of variance, tests for correlations, and regression analyses were used.

**Results:**

Mean age was 51.3 years, 59% were male and 26.3% had ≥12 years of school attendance. Regarding somatic conditions 6.0% were suffering from type 2 diabetes mellitus, 6.9% were affected by new-onset diabetes after transplantation (NODAT), and the mean estimated glomerular filtration rate (eGFR) was 47.7 ml/min/1.73m^2^. The prevalence rates of obesity before and after kidney transplantation were 14.8 and 19.9%, respectively. This represents an increase of 34%. Obesity after transplantation was associated with higher rates of type 2 diabetes mellitus and of NODAT. Additionally, there was an association between increasing pre-transplant as well as post-transplant BMI and decreasing eGFR. Higher age and female sex were associated with higher rates of post-transplant obesity.

**Conclusions:**

Our results suggest that obesity represents a serious problem in KTx patients, especially regarding the association between increasing BMI and decreasing graft functioning (eGFR). However, this aspect is often overlooked and information on effective treatment options for these patients are scarce making further research on this topic necessary.

## Introduction

In the last decades obesity [body mass index (BMI) of more than 30 kg/m^2^] has become a global health problem. It does not only affect the general population but also patients with end-stage kidney disease (ESRD) and patients after kidney transplantation (KTx) (1–4). Of note, obesity is a risk factor for the development and progression of chronic kidney disease (CKD) (5). Due to the low number of post-mortal organ donors, patients have to wait several years for a kidney transplant and depend on dialysis treatment to bridge the time until transplantation (6, 7). In patients undergoing dialysis treatment, the “obesity paradox” has been described: Patients with a higher BMI showed better survival on dialysis (2, 8, 9). This inverse epidemiology does not extend to the post-transplantation period. In the last years four meta-analyses have been published focusing on the impact of obesity on the outcome after kidney transplantation (1–4). While not entirely overlapping the studies hint at higher rates of short-term complications (e.g. delayed graft function and surgical complications) and more adverse long-term complications (e.g. cardiovascular diseases) in KTx patients with obesity. Regarding graft loss controversial results were found (1–4), with some studies suggesting an increased risk for graft loss for high BMI recipients, whereas others showing no significant difference. Above that, it is well known that obesity is associated with hypertension, dyslipidemia, diabetes mellitus, and cardiovascular events, which might not only severely affect the patients' physical health, but also their quality of life (10). However, transplantation still provides a clear survival advantage over dialysis and most obese patients who receive a transplant have better outcomes than those who remain wait-listed (11). Still, they may derive a lower survival benefit from transplantation (12).

Another problem is that patients after organ transplantation seem to be receptive for weight gain (13, 14), especially those already obese at the time of transplantation. There are several possible explanations for this development. On the one hand, an improved physical condition after transplantation may enable weight gain (13). On the other hand, patients after KTx experience fewer restrictions regarding the nutrients and beverages they can consume in comparison to dialysis patients. Above that, the immunosuppressive medication might support weight gain after KTx. While the findings on corticosteroids in maintenance dose and weight gain after KTx seem inconsistent (15), calcineurin inhibitors are known to support new-onset diabetes after transplantation (NODAT) which also supports further weight gain (16).

In conclusion there is ample evidence that obesity negatively affects outcome after KTx. However, in clinical practice little attention is payed to the problem of obesity and weight gain after transplantation. While the potential consequences of obesity after transplantation are well understood, information regarding factors associated with weight gain and obesity after KTx is scarce.

The first aim of our study was to evaluate the course of weight after KTx and to assess the prevalence of post-transplant obesity in a large sample of German KTx patients. There are only few studies available on obesity in German KTx cohorts (13, 17, 18). We expect our findings to be in line with the results of previous studies. However, due to the longer observation period after transplantation in comparison to the other studies, there is a possibility of slightly divergent results.

The second aim of our study was to explore potential associations between weight gain and obesity after transplantation and sociodemographic, medical, psychological, and transplant-specific variables. So far, information on associated variables is inconsistent. Therefore, regarding this aim there was no formal hypothesis due to the exploratory nature of the investigation.

## Methods

### Participants

Participants were recruited within the structured post-transplant care program (KTx360°) at Hannover Medical School (19). All participants received a psychosocial assessment conducted by a medical doctor or psychologist. Above that, participants completed questionnaires assessing sociodemographic, medical, and psychosocial aspects.

To be able to provide answers for the research question of this study, information regarding body weight before transplantation was required. For patients undergoing dialysis treatment before transplantation, the pre-transplant dry weight was taken from the electronic patient charts. For patients who were transplanted preemptively, the body weight immediately before transplantation was used. Patients transplanted at childhood age were excluded from this study as further growth and weight gain are expected to occur in this group. At post-transplant assessment body weight was measured in the transplant center.

Within this ongoing study, 983 KTx patients fitting the inclusion criteria for this substudy were approached between May 2017 and December 2018 at Hannover Medical School. Of those 459 (46.7%) patients participated in the KTx360°study and measured post-transplant heights and weights—mandatory for our substudy—were available of 433 (94.3%) out of the 459 patients.

KTx patients who chose not to participate in the KTx360° study were significantly older, had a longer time since KTx, had more often received an organ from a deceased donor, had a higher rate of diabetes, and a lower rate of anemia compared to the participants. There was no significant difference regarding sex, renal functioning, and hypertension.

The study was approved by the Institutional Ethics Review Board of Hannover Medical School (Number 3464–2017), and all participants gave written informed consent.

### Assessment Instruments

#### Medical Parameters

The estimated glomerular filtration rate (eGFR) at post-transplant assessment and information on the diagnoses of hypertension, renal anemia, and diabetes mellitus were taken from the patient charts. Following the findings of Ekberg et al. (20), the standard of care for KTx patients at Hannover Medical School is a triple-drug immunosuppressive regimen consisting of prednisolone, mycophenolate mofetil, and a calcineurin inhibitor. Body weight and body height were measured using a standardized scale. Patients with a BMI below 25 kg/m^2^ were categorized as under- and normal-weight, patients with a BMI between 25 kg/m^2^ and below 30 kg/m^2^ were graded as overweight and patients with a BMI above 30 kg/m^2^ were rated as obese. For more detailed analyses patients with obesity were divided into three groups based on their BMI: Grade 1 (BMI from 30 kg/m^2^ to below 35 kg/m^2^), grade 2 (BMI from 35 kg/m^2^ to below 40 kg/m^2^), and grade 3 (from BMI 40 kg/m^2^ and above).

#### Socio-Demographic and Donation-Specific Variables

Socio-demographic and donation-specific variables including sex, age, level of education, donation type, time since KTx, and dialysis duration were assessed using a self-report questionnaire. Missing information was taken from the medical records.

#### Symptoms of Depression and Anxiety

Symptoms of anxiety and depression were evaluated with the German version of the Hospital Anxiety and Depression Scale (HADS) (21, 22). The self-report instrument is specifically validated to assess levels of anxiety and depression in patients with somatic comorbidities. There are two subscales “depression” and “anxiety” each consisting of seven items. Each item is scored from 0 to 3, leading to a total score between 0 and 21. Higher scores indicate higher levels of depression or anxiety. Cronbach's α in our sample was 0.86 for depression and 0.81 for anxiety.

#### Statistical Analyses

For each variable descriptive statistics (percentage or mean and standard deviation and median and interquartile range) were calculated for the entire sample and separately for the different BMI categories as described above. Pearson correlations were used to explore the association between the BMI and somatic, sociodemographic, and psychosocial parameters. Kruskal-Wallis tests were used for comparison of continuous variables between the BMI categories. Dunn-Bonferroni *post hoc* tests were conducted for pairwise comparisons of BMI categories. Chi-square tests were used for categorical data. Two linear regression analyses with eGFR as the dependent variable and sex, age, time since transplantation, diabetes mellitus, and either pre-transplant or post-transplant BMI as independent variables were conducted. For all analyses, p < 0.05 was considered statistically significant. All statistical analyses were performed using IBM^®^ Statistical Software Package of Social Science (SPSS^®^, Chicago, IL, USA) version 26.

## Results

### Participants Characteristics

**Figure 1:**
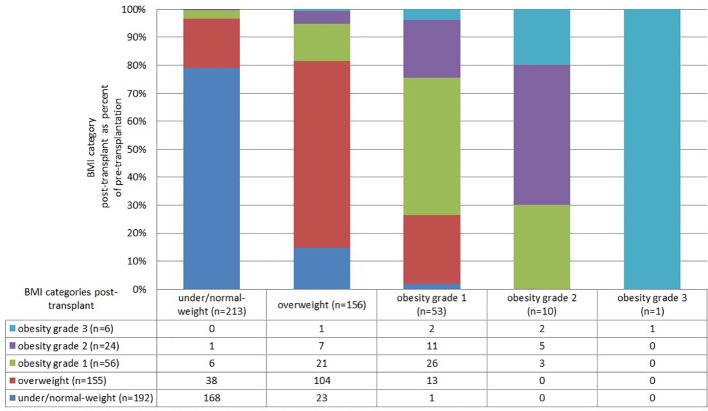
Change in weight category pre- (x-axis) to post-transplantation (y-axis).

Participant characteristics are reported in **Table 1** and **Figure 1**. Our sample consisted of 177 women (40.9%) and 256 men (59.1%). The mean age at post-transplant assessment was 51.3 years (SD 14.1). 26.3% had 12 or more years of school attendance. The mean time since transplantation was 48.5 months; 31.4% were living donor recipients. Regarding diabetes mellitus, 6.7% were diagnosed with type 1, 6.0% with pre-existing type 2 and 6.9% with NODAT. Overall, 21.2% exhibited an eGFR below 30 ml/min/1.73m^2^ at post-transplant assessment, corresponding to a severe reduction of the glomerular filtration rate (23). In our study, prednisolone was part of the medication in 98.6% of the patients, only 1.4% did not receive maintenance cortison therapy. The standard dosing of prednisolone was 5mg/day. This low percentage of patients not receiving cortison therapy is in line with the findings of a previous study conducted at our transplant center [1.1% in Kugler et al. (13)].

**Table 1:** BMI categories at the time of post-transplant assessment (n=433).

Variable	All (n = 433)	Under and normal-weight (n = 192)	Overweight (n = 155)	Obesity Grade 1 (n = 56)	Obesity Grade 2 (n=24)	Obesity Grade 3 (n = 6)	Statistical analyses
Age (years) mean (SD) median (IQR)	51.3 (14.1) 53.0 (20)	49.5 (14.7) 51.0 (22)	54.0 (13.2) 56.0 (17)	50.6 (14.0) 52.0 (21)	52.9 (12.6) 54.5 (16)	43.2 (14.7) 37.5 (29)	**X^2^ = 10.27, df=4, p=0.04**
eGFR (ml/min/1.73m^2^) mean (SD) median (IQR)	46.1 (19.0) 43.1 (24.6)	47.7 (20.5) 43.1 (27.1)	45.6 (17.1) 44.9 (22.3)	44.8 (19.0) 42.2 (24.9)	41.8 (17.4) 43.5 (30.8)	34.3 (20.9) 27.0 (21.5)	X^2^ = 5.65, df=4, p=0.23
Weight change (%) mean (SD) median (IQR)	3.8 (11.5) 2.4 (11.9)	0.4 (10.4)^a,b,c^ 0.8 (10.3)	3.5 (9.0)^a^ 2.4 (11.6)	8.9 (12.3)^b,d^ 5.9 (12.1)	16.7 (10.9)^a,d^ 16.4 (10.8)	24.9 (22.7)^c^ 20.7 (39.0)	**X^2^ = 67.45, df=4, p < 0.001**
Time since transplantation, months mean (SD) median (IQR)	48.5 (49.3) 35.0 (71)	51.7 (52.2) 41.0 (73)	46.0 (47.6) 34 (65)	39.8 (45.5) 22.0 (62)	57.1 (40.8) 63.0 (64)	57.5 (64.4) 32.0 (108)	X^2^ = 5.03, df=4, p=0.28
Time on dialysis, month (n=419) mean (SD) median (IQR)	62.1 (49.8) 54.0 (83)	61.9 (51.7) 53.5 (82)	63.4 (49.1) 58.0 (83)	58.7 (47.1) 48.0 (78)	62.5 (47.6) 45.5 (87)	60.4 (53.9) 42.0 (103)	X^2^ = 0.43, df=4, p=0.98
HADS Depression (n=416) mean (SD) median (IQR) Anxiety (n=415) median (IQR) mean (SD)	4.3 (3.9) 3.0 (5.0) 5.1 (3.9) 4.0 (6.0)	4.1 (3.9) 3.0 (5.0) 5.1 (3.8) 4.0 (5.0)	4.2 (3.8) 3.0 (5.0) 4.9 (3.8) 4.0 (6.0)	5.2 (4.5) 4.0 (6.0) 6.0 (4.2) 5.0 (8.0)	4.9 (3.8) 4.0 (6.0) 4.5 (3.6) 4.0 (4.5)	3.2 (1.5) 3.0 (2.5) 3.4 (2.1) 3.0 (3.0)	X^2^ = 3.91, df=4, p=0.42 X^2^ = 4.58, df=4, p=0.33
Sex, female (%)	40.9 (n=177)	43.8	34.2	37.5	62.5	66.7	**X^2^ = 10.08, df=4, p=0.04**
School attendance ≥ 12 years (%) (n=410)	26.3 (n=108)	32.8	22.0	20.8	16.7	33.3	X^2^ = 7.39, df=4, p=0.12
Living donor recipients (%)	31.4 (n=136)	32.3	31.0	32.1	29.2	16.7	X^2^=.78, df=4, p=0.94
Diabetes mellitus Type II (%)	6.0 (n=26)	2.1	6.5	8.9	25.0	16.7	**X^2^ = 22.686, df=4, p < 0.001**
NODAT (%)	6.9 (n=30)	4.7	6.5	8.9	16.7	33.3	**X^2^ = 11.91, df=4, p=0.02**
eGFR < 30 (%)	21.2 (n=92)	18.8	20.0	25.0	29.2	66.7	**X^2^ = 9.63, df=4, p=0.047**
Change BMI category (%) Obese pre & post Obese pre only Obese post only Never obese	11.5 3.2 8.3 76.9	0 0.5 0 99.5	0 8.4 0 91.6	51.8 0 48.2 0	66.7 0 33.3 0	83.3 0 16.7 0	**X^2^ = 466.27, df=12, p < 0.001**

Different superscripts indicate a significant difference in post-hoc test (Dunn-Bonferroni-Test)

HADS, Hospital Anxiety and Depression Scale; IQR, interquartile range; SD, standard deviation; NODAT, new-onset diabetes after transplantation; eGFR, estimated glomerular filtration rate. Statistically significant results (p < .05, p < .01 and p < .001) are shown in boldface.

### BMI Categories and Changes in BMI

The distribution and changes in BMI groups can be found in **Table 1**. At the time of transplantation, the mean BMI was 25.49 kg/m^2^ (SD 4.28). At post-transplant assessment, the mean BMI was 26.40 kg/m^2^ (SD 5.05). At post-transplant assessment, 19.9% (n=86) of the participants were obese as opposed to 14.8% at the time of transplantation (**Figure 1**). Of those 58.1% had already been obese at the time of transplantation, while 41.9% became obese after transplantation. Only 3.2% of the patients who were obese at the time of transplantation lost enough weight to reach normal-weight or overweight status after transplantation.

The mean weight gain between transplantation and post-transplant assessment was 2.7 kg (SD 8.4) or 3.8% (SD 11.5). Patients with obesity at the time of transplantation (n=64) did not gain significantly more weight than overweight and normal-weight patients (3.2, 1.8, and 2.9 kg, respectively). Nevertheless, of those who were obese at the time of transplantation, n=42 (65.6%) gained 10% or more during the post-transplant period. Patients who were obese at post-transplant assessment reported a mean weight gain of 10 kg or 12.2%. Even within the obese group, there was a positive association between the amount of weight gain and the severity of obesity (**Table 1**) and the correlation between post-transplant BMI and the amount of weight gain was significant (r=0.45, p < 0.001).

### Associations With Sociodemographic and Transplant-Specific Variables

The BMI categories differed significantly with regard to sex distribution (**Table 1**). While only 34.2% of the participants with overweight were female, 66.6% in the participants with obesity grade 3 were female. Age was significantly associated with both the BMI at the time of transplantation and the BMI at post-transplant assessment with older patients having a higher BMI (**Tables 1** and **2**). There was no significant difference regarding the rate of patients with 12 or more years of school attendance between BMI groups.

**Table 2:** Correlations with BMI at the time of transplantation and BMI at post-transplant assessment.

	BMI at time of transplantation*	BMI at post-transplant assessment*
Age	**.25, p < 0.001**	**.10, p = 0.04**
Time on dialysis	.03, p = 0.62	.01, p = 0.77
Time since Tx	—	-.01, p = 0.92
eGFR	**-.23, p < 0.001**	**-.14, p = 0.004**
HADS Anxiety	-.03, p = 0.50	-.00, p = 0.93
HADS Depression	.04, p = 0.44	.06, p = 0.23

BMI, Body Mass Index; HADS, Hospital Anxiety and Depression Scale; Tx, transplantation; eGFR, estimated glomerular filtration rate.

*BMI at the time of transplantation and at post-transplant assessment were highly correlated (r=.82, p < 0.001).

Statistically significant results (p < .05, p < .01 and p < .001) are shown in boldface.

Regarding transplant-specific outcomes time on dialysis, time since transplantation and donation type (living vs. post-mortal donation) were not associated with BMI neither at the time of transplantation nor at post-transplant assessment (**Table 2**).

### Associations With Psychological Symptoms

We found no correlation between the level of symptoms of anxiety or depression measured with the HADS and the BMI at the time of transplantation or at post-transplant assessment (**Tables 1** and **2**).

### Associations With Diabetes Mellitus and Graft Function

Overall, 6.0% of the participants were suffering from pre-existing type 2 diabetes mellitus, while 6.9% developed NODAT. In the BMI categories (**Table 1**), the rate of type 2 diabetes mellitus significantly increased from 2.1% in the under- and normal-weight group to 16.7% in the patients with obesity grade 3. Also, the NODAT rate was lowest in the under- and normal-weight group (4.7%) and highest in the group with obesity grade 3 (33.3%). This difference was statistically significant as well.

Taking a look at the eGFR we found a nonsignificant decrease as the BMI category increased, from 47.7 ml/min/1.73m^2^ in patients with under- and normal-weight to 34.3 ml/min/1.73m^2^ in patients with obesity grade 3 (**Table 1**). However, comparing the rate of patients with an eGFR below 30 ml/min/1.73m^2^, we found a steady and significant increase across BMI categories from 18.8% in patients with under- and normal-weight to 66.7% in patients with obesity grade 3. Additionally, eGFR was significantly and inversely correlated with BMI at the time of transplantation as well as the BMI at post-transplant assessment (**Table 2**). Also, two linear regression analyses revealed that pre-transplant as well as post-transplant BMI were significantly associated with eGFR after adjusting for sex, age, time since transplantation, and the presence of diabetes mellitus (adjusted R-squared approaching 10% in both analyses) (**Table 3**).

Table 3Summary of linear regression analysis with eGFR as dependent variable.

**Table:** 

a) post-transplant BMI					
	**Unstandardized coefficients**		**Standardized coefficient**	**t**	**p**
	**B**	**SE B**	**β**		

Constant	59.55	10.77		5.5.3	**<0.001**
Sex	3.14	1.78	0.08	1.76	0.08
Age (years)	-0.34	0.07	-0.25	-5.09	**<0.001**
Time since transplantation (months)	0.01	0.02	0.04	0.77	0.45
Diabetes mellitus (type 2 or NODAT)	2.08	1.79	0.06	1.16	0.25
BMI post-transplantation	-0.37	0.18	-0.10	-2.07	**0.04**

The model explained 8.3% (adjusted R^2^) of the variance [F(5.427)=8.78, p < 0.001].

Levels of multicollinearity were low with all VIF <1.2.

**Table:** 

b) pre-transplant BMI					
	Unstandardized coefficients		Standardized coefficient	t	p
	B	SE B	β		
Constant	68.67	10.83		6.34	**<0.001**
Sex	3.20	1.77	0.08	1.81	0.07
Age (years)	-0.29	0.07	-0.22	-4.33	**<0.001**
Time since transplantation (months)	0.00	0.02	0.01	0.23	0.82
Diabetes mellitus (type 2 or NODAT)	1.56	1.77	0.04	0.88	0.38
BMI at transplantation	-0.74	0.22	-0.17	-3.38	**0.001**

The model explained 9.8% of the variance [F(5.427)=10.34, p < 0.001].

Levels of multicollinearity were low with all VIF <1.2.

Statistically significant results (p < .05, p < .01 and p < .001) are shown in boldface.

We found no association between the BMI and hypertension, cardiovascular disease, or renal anemia (data not shown).

## Discussion

The frequencies of obesity (BMI > 30 kg/m^2^) in a large German sample of patients before and after kidney transplantation (n=433) were 14.8 and 19.9%, respectively. This is somewhat lower compared to the prevalence of obesity in the German general population [23%; (24)]. The results are in accordance with the results of another German study [17.4% pre-transplant; (18)]. The mean BMI was slightly higher post-transplant compared to pre-transplant (25.49 versus 26.40 kg/m^2^). Overall, we found a significant positive association between pre- and post-transplant BMI (r=0.82) and between post-transplant BMI and the amount of weight gain since transplantation (r=0.45). Thus, increased BMI levels were related to obesity before transplant. Particularly, those patients who presented with higher grades of obesity post-transplant had gained a substantial amount of weight. This result is in line with previous research. Kugler et al. (13) reported a significant increase in BMI in patients after organ transplantation across different organ groups. Significant post-transplant weight gain might be more strongly associated with graft loss or death compared to pre-transplant BMI (25, 26).

In accordance with the second aim of our study we explored the possible association of different variables with weight gain and obesity after KTx. While it can be hypothesized that weight gain and consequently BMI increases with more extended time since transplantation as it does with growing age, we found no statistically significant difference regarding time since transplantation between BMI groups. A possible explanation might be that weight gain occurs predominantly in the first year after transplantation as suggested by Henggeler et al. (27) and Aksoy (14). Henggeler et al. (27) reported that the number of obese patients doubled by one year after transplantation, from 5.6 to 11.4%. In our sample we observed a 34% increase of the rate of obese patients on average 48.5 months after transplantation.

According to the German Organ Transplantation Foundation (DSO) annual report of 2018 (28) type 1 diabetes mellitus was the main diagnosis leading to listing for KTx in 220 of the 2348 (9.4%) cases in Germany. However, when examining obesity, another type of diabetes mellitus is of interest. While not listed as one of the top 10 diagnoses leading to listing for KTx, type 2 diabetes mellitus is common in patients with ESRD and even represents an independent risk factor for the development of chronic kidney disease (CKD) (29). After transplantation, NODAT as another form of diabetes mellitus might occur. We found a strong association between post-transplant BMI categories and type 2 diabetes mellitus as well as NODAT. The causal mechanisms leading to NODAT are not completed understood so far; however, as in type 2 diabetes mellitus obesity is known to be an independent risk factor for its development (29, 30). Our results are thus in line with current literature and support the already described association.

With the threshold dose for developing Cushing's syndrome being at 7.5 mg prednisolone per day, our patients are securely below this threshold and not expected to experience excessive weight gain or other symptoms associated with Cushing's syndrome. Results on corticosteroids in maintenance dose and weight gain after KTx seem inconsistent (15). Most studies show that weight gain in the first year after transplantation seems to be independent of corticosteroid therapy and dosing (13, 31). In patients with rheumatoid arthritis a dose between 5 and 7.5 mg prednisone equivalent per day turned out to be relevant for weight gain over the years (32). Most of our patients were evaluated several years after transplantation. However, as nearly all KTx patients in our sample receive prednisolone and only some of them experience weight gain, it is obvious that there must be other factors contributing to increased weight after KTx.

In our sample, we did not detect an association between BMI and hypertension, cardiovascular disease, and renal anemia, even though there is a well-known association between obesity and hypertension as well as cardiovascular disease. One explanation might be that a high percentage of KTx patients (88.0% in our sample) suffered from hypertension even those who were normal-weight. On the other hand, the percentage of patients with cardiovascular diseases was small (11.8%). As far as we know renal anemia is not known to be associated with BMI. However, further studies on larger samples are required to evaluate the influence of the BMI on somatic conditions in KTx patients.

In addition, we confirmed that obesity is associated with graft function after KTx. We found a small but significant association between both pre- and post-transplant BMI and post-transplant eGFR which was not merely explained by sex, age, time since transplantation, and the presence of diabetes mellitus (type 2 and NODAT). The rate of patients with a reduced eGFR (< 30 ml/min/1.73m^2^) increased across BMI groups and was highest in patients with obesity grade 3. These results are in agreement with the findings reported by others (18, 33, 34) who described a decrease in renal graft functioning as BMI increases within the first year after transplantation. Above that, obesity is known to be an independent risk factor for a decline in kidney functioning, even though pathophysiological mechanisms are not fully understood (26). It has been suggested that poorer postoperative outcomes following solid organ transplantation might be related to obesity-associated adipose tissue inflammation or obesity-associated changes in the intestinal microbiota (35); however, there is still a significant knowledge gap in understanding the mechanisms for the poorer transplant outcomes in patients with obesity.

Regarding sociodemographic variables there was a higher percentage of females in the obese group compared to the other groups. The phenomenon that more men are overweight (BMI ≥ 25 kg/m^2^) but that more women are obese can be found in the general population as well (24). Literature suggests that several different aspects including genetic ones as well as psychosocial ones are responsible for this difference (36). Also, in accordance with the general population, the BMI in our sample of kidney transplant recipients was positively associated with age (24). However, in contrast to findings from the general population (24), we found no statistically significant difference regarding educational level between the BMI groups.

Research suggests that there is an association between obesity and mental disorders, specifically anxiety disorders and depression (37, 38). In our sample, we found no statistically significant difference between BMI groups regarding the self-reported levels of depression and anxiety. Overall, the mean scores were low and well within the normal range which might explain the lack of an association with obesity.

We acknowledge the limitations of our study. First, the retrospective nature of the study made it impossible to adjust for all possible confounding factors. While we included some of the somatic conditions associated with obesity and weight change in our analyses, we were not able to take all possible variables into account. Information on rejection episodes requiring treatment, severe infections and detailed information about immunosuppressive medication, especially cumulative corticosteroid doses, are a few examples of potential confounding factors that were not available for our analyses. Second, we have to take the possibility of a selection bias into account: Even though we were able to evaluate a large sample of kidney transplant recipients from the KTx 360° study (n=433), only roughly half of the KTx patients who met the inclusion criteria for the KTx360° study and were approached participated. However, even though participants and non-participants differed in baseline characteristics, the associations we found between BMI and the examined variables are in line with other studies in German KTx patients using consecutive samples (13, 17, 18). Finally, our data do not allow analyzing the association between obesity and graft failure or recipient mortality.

In conclusion, we were able to show that obesity affects 19.9% of our patients after KTx. This number is slightly lower, but comparable to the general population in Germany. As we hypothesized, it is in line with other studies evaluating KTx patients in Germany. The majority of our patients gained weight after transplantation. In accordance with the second aim of our study we were able to come to the following conclusions: Post-transplant obesity was associated with higher age and female gender and with higher rates of type 2 diabetes mellitus and the development of NODAT. These conditions are known to be able to negatively affect kidney functioning. Above that, we found an association between increasing BMI and decreasing graft functioning (eGFR). Our results are cause for concerns and underline the necessity of obesity management in KTx patients. From a clinical perspective, patients are in need of tailored interventions for weight loss including dietary modification, exercise programs, and behavioral approaches or even bariatric surgery depending on the BMI and comorbidities (39). Although most obese patients selected for transplantation derive a survival benefit, the benefit is significantly lower when BMI is ≥ 40 kg/m^2^ (12). Although there is agreement that obesity affects renal transplant outcomes there is usually only limited support offered to the patients with regard to weight reduction or at least stabilization. Patients might benefit from strategies to prevent weight gain after KTx, particularly when they are already obese pre-transplant. However, information on obesity treatment and prevention in this special group of patients is still scarce (27, 39–41) and further research is required in this field.

## Data Availability Statement

The datasets generated for this study are available on request to the corresponding author.

## Ethics Statement

The studies involving human participants were reviewed and approved by Institutional Ethics Review Board of Hannover Medical School (Number 3464–2017). The patients/participants provided their written informed consent to participate in this study.

## Author Contributions

LP, MS, and MZ designed the KTx360° trial and obtained research funding. LS was essential in the recruitment process of the study. MZ and MN designed this sub-study. ES and AH provided information on measured post-transplant body weight. MZ and MN analyzed the data. MN wrote the first draft of this paper. All authors critically revised the manuscript and read and approved the final version.

## Funding

The study is supported by a grant the Federal Joint Committee of the Federal Republic of Germany under the number 01NVF16009.

## Conflict of Interest

The authors declare that the research was conducted in the absence of any commercial or financial relationships that could be construed as a potential conflict of interest.
